# Genome‐Wide Identification of MYB Genes and Analysis of Their Expression Under Cold Stress Conditions in *Artocarpus heterophyllus*

**DOI:** 10.1002/pld3.70131

**Published:** 2026-04-10

**Authors:** Xiangwei Ma, Pengjin Zhu, Weiyan Ye, Chenxin Yi, Xiuguan Tang, Jianjun Liang, Zhuangmin Wei, Qiqi Song, Hailan Zhou, Shengli Tang

**Affiliations:** ^1^ Guangxi Institute of Subtropical Crops Nanning China; ^2^ Key Laboratory of Quality and Safety Control for Subtropical Fruit and Vegetable, Ministry of Agriculture and Rural Affairs Nanning China; ^3^ Guangxi Key Laboratory of Quality and Safety Control for Subtropical Fruits Nanning Nanning China

**Keywords:** *Artocarpus heterophyllus*, cold stress, expression profile, myeloblastosis (MYB)

## Abstract

Jackfruit ( *Artocarpus heterophyllus* ) thrives in subtropical and tropical areas but is vulnerable to winter cold stress. The myeloblastosis (MYB) transcription factor family plays an important role in plant biological and abiotic stress responses; however, their response to regional cold injury in jackfruit remains unexplored. The study aimed to identify and analyze the expression of 298 MYB genes in jackfruit under cold stress conditions. The study used genome‐wide identification, bioinformatics analysis, and qPCR to analyze the expression of 298 MYB genes in jackfruit under cold stress. Evolutionary tree analysis showed that the AhMYB family members were divided into seven subfamilies. Chromosome mapping results showed that 298 AhMYB family members were unevenly distributed on 27 chromosomes. In addition, analysis of conserved motifs and gene structure showed that members of the AhMYB family located in the same subfamily had similar conserved motifs and gene structure. Collinear analysis identified 1439 duplicated fragments involving AhMYB family members within the species. In addition, the *cis*‐acting elements in the promoters of AhMYB family members are implicated in many aspects of plant growth and development. Transcriptomic analysis of jackfruit under low‐temperature stress showed that the expression patterns of AhMYB family members differed in jackfruit varieties. qPCR analysis further verified this result, confirming that AhMYB family members are involved in the response to low‐temperature stress in jackfruit varieties. These findings provide new insights into the functions of AhMYB family members.

**Abbreviations**
BLAST: Basic Local Alignment Search Tool
CAT: catalase
CDS: coding DNA sequence
ERE: ethylene response elements
FPKM: fragments per kilobase of transcript per million mapped reads
LTR: low‐temperature‐responsive
ROS: reactive oxygen species
SOD: superoxide dismutase
TFs: transcription factors
WGCNA: weighted gene co‐expression network analysis
WT: wild‐type

## 1 Introduction

Transcription factors (TFs) are commonly found in eukaryotes. They form transcription initiation complexes with RNA polymerase and participate in the process of transcription initiation. As one of the largest families of multifunctional TFs in plants, MYB proteins are widely involved in regulating plant growth and development, hormone signal transduction, and stress response at the gene transcription level. MYB TFs have a conserved MYB domain at the N‐terminal, which typically comprises one to four incomplete repeats (R). Each incomplete repeat sequence is the basic unit of the DNA‐binding domain of MYB proteins, consisting of 50–53 amino acid residues and forming a 3α‐helical structure that is favorable for the effective binding of MYB TFs and target DNA. The C‐terminal of MYB protein is highly variable, enabling MYB TFs to perform diverse biological functions. The MYB TF family is divided into four subfamilies based on the number of repeats: 1R‐MYB, R2R3‐MYB, 3R‐MYB, and 4R‐MYB. Most MYB TFs are R2R3‐MYB type, which is also the most studied type, with wide distribution and numerous functions. 1R‐MYB type TFs are the second largest group in the MYB family, usually containing one or part of the repeat sequence, while the 4R‐MYB type is the smallest group, found only in very few plants. Previous studies have shown that MYB TFs play an important role in resistance to cold stress in higher plants. For example, overexpression of R2R3‐type MYB genes can enhance cold stress tolerance in rice ( *Oryza sativa* ) (Yang et al. 2012). Additionally, *Malus baccata* MbMYB4 overexpression in *Arabidopsis* significantly improved the cold resistance of transgenic plants. Under cold stress, transgenic *Arabidopsis thaliana* exhibited increased proline and chlorophyll contents, as well as elevated peroxidase and catalase (CAT) activities, whereas malondialdehyde (MDA) content and relative conductivity significantly decreased (Yao et al. 2022). Tang et al. (2023) revealed that the expression levels of 31 R2R3‐MYB genes increased significantly after cold stress, and transcriptome and weighted gene co‐expression network analysis (WGCNA) showed that SaMYB098, SaMYB015, and SaMYB068 played an important role in the cold resistance process of *Santalum album* . Overexpression of *Pyrus betulaefolia* PbrMYB5 in tobacco (*Nicotiana benthamiana*) increased plant tolerance to low‐temperature stress, whereas its silencing increased the sensitivity of *Pyrus betulaefolia* to low‐temperature stress (Xing et al. 2019). In addition, *Glycine max* GmMYBJ1 expression has been induced by low‐temperature stress. *A. thaliana* overexpressing GmMYBJ1 showed increased tolerance to cold stress compared with wild‐type (WT) plants (Su et al. 2014).

Jackfruit ( *Artocarpus heterophyllus* Lam.) is an evergreen tree belonging to the mulberry family. Also known as wood pineapple, it is native to India and widely distributed in South Asia, Southeast Asia, Oceania, and other places (Ullah and Haque 2008). It thrives in tropical climates and is commonly cultivated in Guangdong, western Yunnan, Guangxi, and Hainan in China (Elevitch and Manner 2006). Jackfruit can be consumed fresh or processed into preserved fruit and jam (Li et al. 2010). In addition, jackfruit seeds and skins have important applications in the food, pharmaceutical and environmental industries, making it an important tropical agroforestry tree (Zeng et al. 2022). Currently, studies have been conducted on the physiology and biochemistry (Nantongo et al. 2022), nutritive chemical composition (Alves Evaristo et al. 2022), and industrial preparation (Maity et al. 2022) and applications (Annzia et al. 2019) of jackfruit. In addition, progress has been made in gene transcription, expression, and germplasm resources characterization. Jackfruit is adapted to hot and moist tropical climates, with temperature being the key factor affecting its growth and development. When temperatures are lower than 5°C–7°C, jackfruit trees are susceptible to flower and fruit drop (Song et al. 2021). Frequent cold damage in winter has become one of the important factors limiting the development of the jackfruit industry. The identification of superior cold‐resistant gene resources, combined with modern molecular breeding methods, can enhance the selection efficiency of superior cold‐resistant varieties and provide important guidance for their improvement and breeding (Du et al. 2023).

MYB genes have been widely reported in higher plants, but the genome‐wide identification of MYB gene family members in jackfruit and their expression patterns under abiotic stress have not been fully elucidated. In this study, 298 AhMYB genes were identified using the reported genome of “S10” jackfruit (Lin et al. 2022). The basic physicochemical properties, phylogenetic relationships, conserved motifs, gene structure, chromosome localization, and collinearity of these genes were analyzed, and their expression patterns in different jackfruits under cold stress were evaluated. The results of this study enhance our understanding of AhMYB and provide insights for breeding novel cold stress‐resistant jackfruit strains.

## 2 Materials and Methods

### 2.1 Plant Material

The experimental material included a local Guangxi jackfruit strain (GX) and an introduced Thai jackfruit strain (THA), both preserved in the germplasm resource nursery of the Guangxi Institute of Subtropical Crops, located at 108°20′34.8″ E and 2°53′56.4″ N. The study was conducted under natural conditions (February 19–24, 2022), following a period of low‐temperature stress in Nanning City, where daily minimum and maximum temperatures reached 3°C and 15°C, respectively. The minimum and maximum daily temperatures at the jackfruit crown were 3.5°C and 14°C, respectively. The leaves of both strains were collected (counting the first leaf on each branch from top to bottom) and stored at −80°C for RNA extraction. Three biological replicates were set up for each experiment.

### 2.2 Identification and Physicochemical Properties of MYB Gene Family in *A. heterophyllus*

The “S10” jackfruit genome (Lin et al. 2022) was downloaded from the National Center for Biotechnology Information (NCBI) Sequence Read Archive (Schoch et al. 2020) under accession numbers PRJNA788174 and PRJNA791757. WRKY gene family sequences of *A. thaliana* were downloaded from TAIR (Swarbreck et al. 2007) (https://www.arabidopsis.org/index.jsp) and used as probe sequences (E < 0.001) for Basic Local Alignment Search Tool (BLAST) analysis. The MYB conserved domain (PF00249) was retrieved from Pfam (http://pfam.xfam.org/) for further annotation of jackfruit MYB gene family members. The physicochemical properties of jackfruit WRKY gene family members were analyzed using the Protein Parameter Calculation function in TBtools (Chen et al. 2020).

### 2.3 Phylogenetic Tree Analysis

The phylogenetic tree of the jackfruit MYB family was constructed using the neighbor‐joining method in MEGAX (Mega 2018) with 1000 bootstrap replicates. The tree was visually enhanced using iTOL (Letunic and Bork 2021) (https://itol.embl.de).

### 2.4 Conserved Motif and Gene Structure Analysis

The conserved motifs within the MYB gene family were analyzed using MEME (Letunic and Bork 2021) (http://meme‐suite.org/tools/meme). The jackfruit genome GFF3 file was used for gene structure reference. Visualization was performed using the Gene Structure Display Server function in TBtools with default parameters.

### 2.5 Chromosomal Mapping and Collinearity Analysis

The chromosomal locations of jackfruit MYB gene family members were visualized using the “Gene Location Visualization from GTF/GFF function” in TBtools based on the jackfruit genome GFF3 file. Collinear gene pairs within the AhMYB family were identified using the “One Step MCScanX” function in TBtools, and the results were visualized using the Advanced Circos feature of TBtools software.

### 2.6 Analysis of *cis*‐Acting Elements of MYB Gene Family Promoter in *A. heterophyllus*

The 2000‐bp upstream nucleotide sequences of jackfruit MYB gene family members were extracted using TBtools. Promoter cis‐acting elements were identified using PlantCARE (http://bioinformatics.psb.ugent.be/webtools/plantcare/html/), and the results were visualized using TBtools.

### 2.7 Expression Pattern Analysis of MYB Gene Family Under Low‐Temperature Stress in *A. heterophyllus*

Fragments per kilobase of transcript per million mapped reads (FPKM) values of the AhMYB gene in GX and THA jackfruit were extracted from the published transcriptome data of different varieties under low‐temperature stress, and heat maps were constructed using the “HeatMap” function in TBtools. The total RNA of GX and THA jackfruit was extracted using a HiScript Q RT SuperMix for qPCR (+gDNA wiper) kit (Novizan, Nanjing, China). cDNA was synthesized using a ChamQ SYBR qPCR Master Mix kit (Novizan), diluted tenfold, and used as an amplification template. Quantitative real‐time PCR (qPCR) was conducted using a QuantStudio 5 Real‐time PCR instrument (Thermo Fisher Scientific, Waltham, MA, USA; Table 1), and Tubulin was used as the reference gene for the qPCR of different varieties under low‐temperature stress. The reaction procedure was as follows: predenaturation at 95°C for 30 s, denaturation at 95°C for 10 s, annealing at 60°C for 30 s, and extension at 72°C for 30 s. The process consisted of 50 cycles repeated thrice. The relative expression of the AhMYB gene was calculated using the 2 − ∆∆Ct method.

**TABLE 1:** Basic parameters analysis of AhMYB gene family.

Gene name	Gene ID	Number of amino acid	Molecular weight (kDa)	Theoretical pI	Instability index	Aliphatic index	Grand average of hydropathicity
AhMYB1	AHE.Chr01.1108	82	9.25	9.57	73.47	51.22	−1.08
AhMYB2	AHE.Chr01.514	352	39.22	5.82	55.5	80.45	−0.523
AhMYB3	AHE.Chr01.701	376	41.19	6.12	44.42	67.02	−0.595
AhMYB4	AHE.Chr01.752.2	294	32.2	9.65	35.64	88.27	−0.451
AhMYB5	AHE.Chr01.857	324	34.56	8.92	59.65	68.33	−0.442
AhMYB6	AHE.Chr02.352	121	13.96	8.3	55.6	49.17	−1.26
AhMYB7	AHE.Chr02.660	330	35.65	8.75	60.09	70.91	−0.446
AhMYB8	AHE.Chr02.818	407	44.85	8.55	57.32	61.55	−0.889
AhMYB9	AHE.Chr03.1146	314	34.69	9.28	42.5	65.22	−0.474
AhMYB10	AHE.Chr03.278	842	93.74	5.11	53.17	68.8	−0.606
AhMYB11	AHE.Chr03.345	269	29.63	6.59	48.38	62.38	−0.653
AhMYB12	AHE.Chr03.390	275	30.29	9.71	57.27	59.6	−0.709
AhMYB13	AHE.Chr03.440	288	31.63	7.65	49.33	66.56	−0.788
AhMYB14	AHE.Chr03.491	263	30.35	5.48	51.32	78.17	−0.871
AhMYB15	AHE.Chr03.492	321	36.05	8.84	36.55	78.69	−0.672
AhMYB16	AHE.Chr03.563	765	84.29	6.07	52.47	58.48	−0.872
AhMYB17	AHE.Chr03.725	326	37.09	8.99	37.31	73.87	−0.822
AhMYB18	AHE.Chr04.1172	491	53.68	6.71	60.91	61.3	−0.858
AhMYB19	AHE.Chr04.1351	327	35.99	9.38	42.75	64.46	−0.513
AhMYB20	AHE.Chr04.1431	330	36.82	8.67	54.42	65.7	−0.646
AhMYB21	AHE.Chr04.311	966	107.54	5.27	48.82	69.61	−0.621
AhMYB22	AHE.Chr04.420	269	29.57	6.7	40.47	62.75	−0.657
AhMYB23	AHE.Chr04.489	304	30.09	9.58	48.77	65.46	−0.61
AhMYB24	AHE.Chr04.526	300	33.02	7.63	56.45	64.9	−0.717
AhMYB25	AHE.Chr04.566	277	32.21	5.57	38.02	75.31	−0.848
AhMYB26	AHE.Chr04.567	337	38.06	8.9	44.2	73.8	−0.739
AhMYB27	AHE.Chr04.885	335	38.03	8.66	37.95	68.09	−0.944
AhMYB28	AHE.Chr04.945	73	8.91	6.11	91.91	77.53	−0.556
AhMYB29	AHE.Chr04.947	73	8.91	6.11	91.91	77.53	−0.556
AhMYB30	AHE.Chr05.1006	183	20.84	5.04	62.69	67.7	−0.634
AhMYB31	AHE.Chr05.1086	419	40.03	8.07	46.8	72.63	−0.591
AhMYB32	AHE.Chr05.1460	473	51.49	7.02	61.6	57.36	−0.633
AhMYB33	AHE.Chr05.1769	326	36.68	6.38	41.42	69.75	−0.675
AhMYB34	AHE.Chr05.379	233	26.79	9.71	57.55	67.85	−0.793
AhMYB35	AHE.Chr06.1138	454	49.47	6.99	58.89	59.36	−0.668
AhMYB36	AHE.Chr06.1482	312	35.28	5.95	52.17	68.14	−0.724
AhMYB37	AHE.Chr06.1496	366	41.12	7.55	43.15	75.14	−0.486
AhMYB38	AHE.Chr06.370	387	43.33	5.44	47.88	67.65	−0.758
AhMYB39	AHE.Chr06.481	625	68.56	5.41	43.92	79.28	−0.463
AhMYB40	AHE.Chr06.673	257	29.65	8.19	59.93	69.84	−0.796
AhMYB41	AHE.Chr07.117	380	42.77	8.03	51.76	68.5	−0.614
AhMYB42	AHE.Chr07.1256	363	41.27	6.1	43.34	77.11	−0.707
AhMYB43	AHE.Chr07.1342	158	18.05	9.3	76.1	54.43	−1.169
AhMYB44	AHE.Chr07.1457	312	34.55	8.91	52.9	78.59	−0.545
AhMYB45	AHE.Chr07.1463	293	33.22	8.93	40.28	76.55	−0.689
AhMYB46	AHE.Chr07.2079	787	83.82	4.96	49.12	71.26	−0.682
AhMYB47	AHE.Chr07.2321	246	26.42	8.67	42.03	55.57	−0.661
AhMYB48	AHE.Chr07.2410	1092	121.6	5.36	58.27	66.08	−0.589
AhMYB49	AHE.Chr07.425	87	10.01	6.73	55.42	63.91	−0.893
AhMYB50	AHE.Chr07.441	85	9.5	9.05	60.51	74.59	−0.642
AhMYB51	AHE.Chr07.593.1	687	75.77	6.03	37.44	74.18	−0.592
AhMYB52	AHE.Chr07.623	373	42.69	5.08	53.55	69.33	−0.857
AhMYB53	AHE.Chr07.731	310	33.89	9.27	59.27	74.87	−0.555
AhMYB54	AHE.Chr07.863	401	44.24	5.72	54.8	73.49	−0.591
AhMYB55	AHE.Chr07.872	334	37.19	7.08	48.81	68.08	−0.642
AhMYB56	AHE.Chr07.897	311	34.32	9.69	46.89	63.02	−0.584
AhMYB57	AHE.Chr07.972	302	34.35	9.07	45.41	74.93	−0.867
AhMYB58	AHE.Chr07.975	203	23.31	9.56	55.66	72.07	−0.893
AhMYB59	AHE.Chr08.1057	345	39.15	6.2	47.75	81.42	−0.587
AhMYB60	AHE.Chr08.1093	377	43.05	9.24	58.53	57.24	−0.942
AhMYB61	AHE.Chr08.1204	231	26.81	9.39	44.83	99.61	−0.545
AhMYB62	AHE.Chr08.1363	309	34.57	9.29	55.84	71.75	−0.664
AhMYB63	AHE.Chr08.1368	70	7.91	9.64	29.84	66.86	−0.704
AhMYB64	AHE.Chr08.1369	174	19.88	8.63	58.38	78.45	−0.7
AhMYB65	AHE.Chr08.1650	724	77.38	5.18	43.33	68.29	−0.731
AhMYB66	AHE.Chr08.2021	261	28.83	8.98	53.75	57.28	−0.759
AhMYB67	AHE.Chr08.2134	1043	115.14	5.28	60.27	62.21	−0.682
AhMYB68	AHE.Chr08.2364	314	33.99	6.6	49.26	76.66	−0.533
AhMYB69	AHE.Chr08.301	100	11.17	9.26	52.89	76.1	−0.632
AhMYB70	AHE.Chr08.315	94	10.93	7.99	56.37	71.7	−0.902
AhMYB71	AHE.Chr08.532	383	43.59	4.81	45.72	69.79	−0.845
AhMYB72	AHE.Chr08.668	309	33.76	9.13	59.36	75.15	−0.541
AhMYB73	AHE.Chr08.741	393	43.59	6.42	50.84	70.28	−0.694
AhMYB74	AHE.Chr08.752	331	36.93	8.34	48.91	70.73	−0.641
AhMYB75	AHE.Chr08.783	354	39.02	10.14	46.34	61.41	−0.62
AhMYB76	AHE.Chr08.869	308	35.22	8.94	46.69	71.56	−0.894
AhMYB77	AHE.Chr08.874	202	23.42	9.11	61.08	72.92	−0.968
AhMYB78	AHE.Chr09.1449	398	44.32	5.93	58.59	63.47	−0.908
AhMYB79	AHE.Chr09.1579	95	10.6	9.22	67.31	54.42	−0.98
AhMYB80	AHE.Chr09.1585.1	103	11.53	7.92	60.54	70.1	−0.762
AhMYB81	AHE.Chr09.170	528	58.02	4.89	55.67	70.38	−0.746
AhMYB82	AHE.Chr09.1996	342	37.82	6.51	40.21	66.7	−0.813
AhMYB83	AHE.Chr09.2109	304	34.66	6.46	50.52	62.83	−0.852
AhMYB84	AHE.Chr09.2112	270	31.29	8.75	52.25	71.85	−0.797
AhMYB85	AHE.Chr09.361	267	29.91	9.07	48.58	73.48	−0.656
AhMYB86	AHE.Chr09.580	274	31.44	5.88	56.38	66.24	−0.782
AhMYB87	AHE.Chr09.6	522	57.26	4.74	53.1	70.8	−0.743
AhMYB88	AHE.Chr10.106	431	48.91	5.52	40.25	72.16	−0.815
AhMYB89	AHE.Chr10.1436	95	10.63	9.19	66.04	54.42	−0.998
AhMYB90	AHE.Chr10.2043	346	38.52	5.78	45.09	66.47	−0.839
AhMYB91	AHE.Chr10.264	266	29.72	8.61	46.35	70.08	−0.698
AhMYB92	AHE.Chr10.500	242	27.57	8.98	57.53	65.29	−0.73
AhMYB93	AHE.Chr10.508	265	30.45	9.12	55.42	70	−0.74
AhMYB94	AHE.Chr10.572	493	54.25	5.09	64.37	64.26	−0.749
AhMYB95	AHE.Chr10.67	513	56.61	4.91	57.39	66.14	−0.76
AhMYB96	AHE.Chr10.87	431	49.02	5.45	43.55	71.69	−0.822
AhMYB97	AHE.Chr11.1004	307	34.35	9.47	51.61	58.14	−0.723
AhMYB98	AHE.Chr11.1025	473	52.66	5.65	51.71	67.04	−0.782
AhMYB99	AHE.Chr11.1311	495	54.79	7.71	46.81	61.7	−0.76
AhMYB100	AHE.Chr11.184	366	41.18	5.83	48.34	69.75	−0.699
AhMYB101	AHE.Chr11.1847	347	38.86	7.64	46.53	67.69	−0.862
AhMYB102	AHE.Chr11.362	264	30.14	9.47	36.62	71.36	−0.655
AhMYB103	AHE.Chr11.648	793	91.65	7.76	36.63	49.75	−1.523
AhMYB104	AHE.Chr11.657	476	54.01	6.85	64.3	69.58	−0.792
AhMYB105	AHE.Chr11.980	329	35.86	5.84	43.66	69	−0.614
AhMYB106	AHE.Chr12.1020_AHE.Chr12.1021	310	34.57	9.54	55.56	60.06	−0.734
AhMYB107	AHE.Chr12.1287	488	53.49	7.74	50.91	66.19	−0.722
AhMYB108	AHE.Chr12.1296	488	53.49	7.74	50.91	65	−0.735
AhMYB109	AHE.Chr12.15	254	28.97	6.92	40.7	69.13	−0.919
AhMYB110	AHE.Chr12.184	322	36.12	5.15	49.05	67.8	−0.766
AhMYB111	AHE.Chr12.2089	325	36.49	5.11	51.12	74.12	−0.699
AhMYB112	AHE.Chr12.401	966	108.86	5.45	52.47	71.51	−0.934
AhMYB113	AHE.Chr12.404	270	30.48	9.36	44.01	70.19	−0.581
AhMYB114	AHE.Chr12.599	422	47.24	6.46	52.07	54.34	−0.792
AhMYB115	AHE.Chr12.634	543	61.24	5.5	59.05	71.2	−0.658
AhMYB116	AHE.Chr12.827	307	35.01	5.36	57.23	59.06	−0.861
AhMYB117	AHE.Chr13.1074	319	34.21	9.2	67.76	69.12	−0.45
AhMYB118	AHE.Chr13.1123	251	29.01	8.39	47.31	77.65	−0.831
AhMYB119	AHE.Chr13.586	381	42.51	8.26	71.25	55.83	−1.005
AhMYB120	AHE.Chr13.919	401	43.56	6.12	43.74	68.88	−0.505
AhMYB121	AHE.Chr13.931	501	55.12	6.54	54.82	60.5	−0.576
AhMYB122	AHE.Chr13.935	349	39.32	6.23	41.66	70.92	−0.903
AhMYB123	AHE.Chr14.1095	323	36.37	6.26	62.99	71.61	−0.638
AhMYB124	AHE.Chr14.1200	338	38.76	6.8	46.38	73.61	−0.696
AhMYB125	AHE.Chr14.1270	678	73.06	6.89	50.47	84.65	−0.287
AhMYB126	AHE.Chr14.396	329	35.43	9.02	67.76	64.98	−0.559
AhMYB127	AHE.Chr14.560	510	56.28	6.16	53.55	60.8	−0.605
AhMYB128	AHE.Chr14.580	422	45.42	6.27	43.07	65.55	−0.468
AhMYB129	AHE.Chr14.967.1	248	27.88	8.91	38.95	83.02	−0.728
AhMYB130	AHE.Chr15.134	412	45.67	5.63	51.45	61.07	−0.831
AhMYB131	AHE.Chr15.1459	293	31.11	6.84	53.05	58.29	−0.695
AhMYB132	AHE.Chr15.66	350	39.5	6.49	52.09	68.29	−0.678
AhMYB133	AHE.Chr15.822	303	34.41	6.07	46.1	74.26	−0.493
AhMYB134	AHE.Chr15.923	281	32.91	9.13	66.4	69.47	−0.905
AhMYB135	AHE.Chr16.1080	310	33.47	5.81	44.02	85.68	−0.259
AhMYB136	AHE.Chr16.121	673	73.49	5.65	39.59	80.09	−0.441
AhMYB137	AHE.Chr16.131	376	42.19	6.11	48.48	62.74	−0.924
AhMYB138	AHE.Chr16.1463	293	31.24	6.55	52.44	63.58	−0.607
AhMYB139	AHE.Chr16.1629	311	33.89	6.52	66.23	57.4	−0.745
AhMYB140	AHE.Chr16.1647	310	33.84	9.5	37.83	90	−0.342
AhMYB141	AHE.Chr16.1710	394	43.02	5.79	43.54	67.13	−0.593
AhMYB142	AHE.Chr16.779	287	32.59	6.01	52.55	60.49	−0.763
AhMYB143	AHE.Chr16.933	274	32.05	9.13	68.12	73.72	−0.856
AhMYB144	AHE.Chr17.1290	289	33.49	7.15	56.41	67.82	−0.82
AhMYB145	AHE.Chr17.1291	344	38.31	5.76	54.9	71.72	−0.461
AhMYB146	AHE.Chr17.204	376	41.77	9.12	43.96	65.59	−0.789
AhMYB147	AHE.Chr17.390	589	64.73	8.67	58.31	62.43	−0.742
AhMYB148	AHE.Chr17.999	335	36.12	8.23	62.62	56.75	−0.696
AhMYB149	AHE.Chr18.1392	590	64.87	8.31	55.03	65.64	−0.713
AhMYB150	AHE.Chr18.1495	312	35.27	9.13	49.65	59.97	−0.771
AhMYB151	AHE.Chr18.232	70	7.83	5.6	72.19	82.14	−0.597
AhMYB152	AHE.Chr18.373	602	67.07	8.17	51.47	66.76	−0.65
AhMYB153	AHE.Chr18.452	89	10.69	6.61	73.1	62.58	−1.078
AhMYB154	AHE.Chr18.769	326	35.45	6.83	51.81	61.32	−0.624
AhMYB155	AHE.Chr18.8	325	36.57	5.11	51.25	73.82	−0.719
AhMYB156	AHE.Chr18.930	272	30.82	8.79	34.9	72.79	−0.767
AhMYB157	AHE.Chr19.1337	256	29.58	9.15	55.87	69.77	−0.792
AhMYB158	AHE.Chr19.1375	704	80.72	5.36	58.79	70.81	−0.745
AhMYB159	AHE.Chr19.173	398	45.73	5.11	52.28	66.58	−0.954
AhMYB160	AHE.Chr19.29	305	34.47	5.41	64.9	70.62	−0.751
AhMYB161	AHE.Chr19.49	381	42.56	5.95	56.98	62.23	−0.664
AhMYB162	AHE.Chr19.505	418	46.88	6.62	43.5	62.56	−0.592
AhMYB163	AHE.Chr19.556	432	50.04	7.23	46.22	65.83	−0.841
AhMYB164	AHE.Chr19.571	319	36.19	5.93	73.69	65.02	−0.716
AhMYB165	AHE.Chr19.580	134	15.92	9.55	49.46	75.67	−0.806
AhMYB166	AHE.Chr19.664	296	32.74	9.27	50.62	82.43	−0.516
AhMYB167	AHE.Chr19.675	318	34.49	6.56	49.88	73.24	−0.583
AhMYB168	AHE.Chr19.739	318	34.6	6.56	50.62	73.24	−0.596
AhMYB169	AHE.Chr19.862	352	39.09	6.62	48.58	68.27	−0.774
AhMYB170	AHE.Chr19.879	340	37.01	5.95	53.29	64.82	−0.729
AhMYB171	AHE.Chr19.880	442	48.2	6.25	47.3	70.88	−0.651
AhMYB172	AHE.Chr19.881	349	39.29	6.03	51.83	69.37	−0.725
AhMYB173	AHE.Chr20.1240	556	62.27	8.78	52.68	68.54	−0.535
AhMYB174	AHE.Chr20.1252	256	29.45	7.67	53.24	67.46	−0.832
AhMYB175	AHE.Chr20.1270	297	33.67	8.28	63.44	63.37	−0.733
AhMYB176	AHE.Chr20.1303.1	327	35.79	6.97	64.65	62.02	−0.689
AhMYB177	AHE.Chr20.1474	1383	155.31	8.04	54.15	71.56	−0.786
AhMYB178	AHE.Chr20.180	399	45.89	5.23	53.53	67.64	−0.962
AhMYB179	AHE.Chr20.494	418	46.82	6.37	43.67	64.21	−0.594
AhMYB180	AHE.Chr20.555	456	52.5	6.11	48.63	63.27	−0.858
AhMYB181	AHE.Chr20.595	208	23.73	7.03	49.96	64.18	−0.739
AhMYB182	AHE.Chr20.654	370	41.14	6.15	51.44	79.11	−0.484
AhMYB183	AHE.Chr20.671	312	33.84	6.56	50.27	77.15	−0.518
AhMYB184	AHE.Chr20.797	353	39.13	6.46	49.6	68.92	−0.793
AhMYB185	AHE.Chr20.888	426	47.07	6.09	47.5	76.95	−0.574
AhMYB186	AHE.Chr21.1002	489	53.83	5.79	59.09	65.03	−0.817
AhMYB187	AHE.Chr21.1104	134	15.21	6.75	38.42	70.75	−0.504
AhMYB188	AHE.Chr21.1561	237	27.28	9.11	60.54	80.93	−0.645
AhMYB189	AHE.Chr21.181	380	41.93	5.46	64.67	69.82	−0.533
AhMYB190	AHE.Chr21.293	275	31.26	6.09	45.67	63.42	−0.904
AhMYB191	AHE.Chr21.362	464	51.11	6.45	64.82	61.36	−0.673
AhMYB192	AHE.Chr21.420	388	42.91	6.28	48.5	63.66	−0.731
AhMYB193	AHE.Chr21.529	509	57.55	9	42.91	66.27	−0.927
AhMYB194	AHE.Chr21.539	230	26.59	7.32	50.52	68.74	−0.905
AhMYB195	AHE.Chr21.666	229	26.74	8.41	61.03	60.44	−1.179
AhMYB196	AHE.Chr21.807	371	40.49	6.6	51.38	63.15	−0.772
AhMYB197	AHE.Chr21.853	372	42.12	9.32	65.58	76.85	−0.657
AhMYB198	AHE.Chr21.943	354	39.78	5.83	54.34	80.76	−0.636
AhMYB199	AHE.Chr21.950	225	25.91	4.82	60.84	41.2	−1.004
AhMYB200	AHE.Chr21.953	286	32.75	5.66	54.6	60.35	−0.826
AhMYB201	AHE.Chr22.1327	356	39.66	5.27	52.41	84.41	−0.461
AhMYB202	AHE.Chr22.1336	274	31.56	5.01	63.82	53.07	−0.992
AhMYB203	AHE.Chr22.1337	302	34.26	5.44	62.72	63.01	−0.708
AhMYB204	AHE.Chr22.1502	343	38.23	9.53	49.53	69.48	−0.808
AhMYB205	AHE.Chr22.1564	73	8.39	4.66	47.98	53.56	−0.885
AhMYB206	AHE.Chr22.1869	174	20.17	9.69	47.64	68.85	−0.845
AhMYB207	AHE.Chr22.260	301	34.8	5.58	42.97	67.01	−0.891
AhMYB208	AHE.Chr22.338	505	57.19	9.18	39.62	65.23	−0.923
AhMYB209	AHE.Chr22.344	232	26.72	8	45.24	70.69	−0.866
AhMYB210	AHE.Chr22.478	52	6.24	6.29	72.71	99.42	−0.19
AhMYB211	AHE.Chr22.479	284	31.24	4.96	69.2	70.7	−0.413
AhMYB212	AHE.Chr22.558_AHE.Chr22.559	464	51.42	6.62	64.06	60.73	−0.745
AhMYB213	AHE.Chr22.574	508	55.35	7.87	60.64	62.32	−0.691
AhMYB214	AHE.Chr22.607	382	42.11	6.16	55.23	62.62	−0.779
AhMYB215	AHE.Chr22.643	380	41.59	5.96	51.16	62.45	−0.853
AhMYB216	AHE.Chr22.835	325	36.36	4.72	53.46	76.18	−0.601
AhMYB217	AHE.Chr22.991	246	28.49	6.66	56.04	59.07	−1.076
AhMYB218	AHE.Chr23.1017	79	9.14	4.47	62.52	54.43	−0.844
AhMYB219	AHE.Chr23.1208	374	42.33	6.1	43.56	67.78	−0.73
AhMYB220	AHE.Chr23.1220	139	15.57	5.24	52.82	89.21	−0.246
AhMYB221	AHE.Chr23.184	245	27.88	6.37	53.69	69.63	−0.8
AhMYB222	AHE.Chr23.2116	460	51.62	9	55.46	75.83	−0.642
AhMYB223	AHE.Chr23.500	112	12.75	4.57	44.94	48.84	−0.883
AhMYB224	AHE.Chr23.513	206	23.76	9.64	58.86	70.53	−0.923
AhMYB225	AHE.Chr23.515	386	43.24	6.08	54.76	70.75	−0.769
AhMYB226	AHE.Chr23.524.2	966	107.12	6.27	51.91	80.62	−0.412
AhMYB227	AHE.Chr23.631	362	39.97	6.44	58.15	60.94	−0.663
AhMYB228	AHE.Chr23.652	282	30.37	8.13	62.42	49.5	−0.781
AhMYB229	AHE.Chr24.1100	79	9.13	4.61	75.43	51.9	−1.014
AhMYB230	AHE.Chr24.1232	377	42.76	6.34	44.42	66.76	−0.729
AhMYB231	AHE.Chr24.1250	276	31.56	4.98	56.8	65	−0.829
AhMYB232	AHE.Chr24.1552	366	41.29	9.72	49.29	75.66	−0.746
AhMYB233	AHE.Chr24.170	322	37.16	7.67	54.33	73.57	−0.733
AhMYB234	AHE.Chr24.2232	403	45.46	8.29	48.47	71.14	−0.679
AhMYB235	AHE.Chr24.259	319	34.29	6.21	45.35	74.29	−0.445
AhMYB236	AHE.Chr24.516	238	27.48	6.01	55.57	69.2	−0.83
AhMYB237	AHE.Chr24.517	289	32.89	6.84	45.36	67.85	−0.816
AhMYB238	AHE.Chr24.527	378	42.15	6.24	54.47	67.09	−0.726
AhMYB239	AHE.Chr24.538	232	27.05	5.79	57.5	65.17	−1.081
AhMYB240	AHE.Chr24.539	222	25.5	5.78	61.09	66.71	−0.862
AhMYB241	AHE.Chr24.540	289	32.93	6.84	46.85	69.2	−0.823
AhMYB242	AHE.Chr24.548	206	23.85	9.35	54.41	68.2	−0.912
AhMYB243	AHE.Chr24.551	378	42.18	6.21	54.07	66.06	−0.733
AhMYB244	AHE.Chr24.557	662	72.55	5.9	50.79	67.84	−0.644
AhMYB245	AHE.Chr24.593	380	42.31	6.63	61.21	85.68	−0.393
AhMYB246	AHE.Chr24.735	361	38.79	6.54	57.4	61.88	−0.671
AhMYB247	AHE.Chr25.1183	423	47.59	5.97	58.29	63.43	−0.8
AhMYB248	AHE.Chr25.1250	423	47.76	6.13	58.97	62.51	−0.822
AhMYB249	AHE.Chr25.134	299	32.96	8.47	39.8	66.82	−0.567
AhMYB250	AHE.Chr25.215	485	54.26	8.57	52.18	70.56	−0.912
AhMYB251	AHE.Chr25.237	485	54.37	8.57	52.18	69.55	−0.912
AhMYB252	AHE.Chr25.310	272	30.63	6.9	50.24	99.26	−0.246
AhMYB253	AHE.Chr25.38	360	38.71	8.72	37.05	77.47	−0.476
AhMYB254	AHE.Chr25.57	273	31.43	8.82	41.21	69.63	−0.858
AhMYB255	AHE.Chr26.1178.2	489	54.52	7.17	52.58	73.15	−0.716
AhMYB256	AHE.Chr26.140	281	30.72	8.33	43.19	75.62	−0.391
AhMYB257	AHE.Chr26.1460	338	35.45	6.5	54.64	64.23	−0.487
AhMYB258	AHE.Chr26.1510	405	44.33	9.51	58.12	62.4	−0.728
AhMYB259	AHE.Chr26.1539	290	31.42	7.76	49.48	67.62	−0.834
AhMYB260	AHE.Chr26.24	446	48.69	6.65	36.13	67.58	−0.659
AhMYB261	AHE.Chr26.251	340	37.31	8.35	57.89	63.71	−0.644
AhMYB262	AHE.Chr26.285	249	28.94	4.8	66.94	57.23	−0.988
AhMYB263	AHE.Chr26.41	144	16.73	9.84	54.3	87.36	−0.436
AhMYB264	AHE.Chr26.691	411	46.18	6.06	62.85	63.84	−0.775
AhMYB265	AHE.Chr27.1255	1384	155.44	7.84	54.71	71.79	−0.782
AhMYB266	AHE.Chr27.140	82	9.65	7.7	30.12	77.2	−0.776
AhMYB267	AHE.Chr27.142	312	35.49	6.73	50.74	64.68	−0.785
AhMYB268	AHE.Chr27.1474	406	45.02	6.52	36.65	80.99	−0.526
AhMYB269	AHE.Chr27.1540	557	61.114	5.08	41.38	77.04	−0.464
AhMYB270	AHE.Chr27.1568	259	28.74	9.1	65.13	73.94	−0.809
AhMYB271	AHE.Chr27.1785	357	40.93	7.23	50.97	64.45	−0.828
AhMYB272	AHE.Chr27.1793.2	371	41.31	8.53	46.7	68.63	−0.69
AhMYB273	AHE.Chr27.1794	362	40.29	8.53	48.03	68.18	−0.698
AhMYB274	AHE.Chr27.299	97	11.95	6.85	74.26	64.33	−0.982
AhMYB275	AHE.Chr27.571	369	40.46	9.44	49.5	64.15	−0.681
AhMYB276	AHE.Chr27.65	326	35.81	9.38	53.05	64.66	−0.647
AhMYB277	AHE.Chr27.725	409	45.46	7.33	49.42	72.47	−0.86
AhMYB278	AHE.Chr27.739	264	28.44	8.27	50.89	75.11	−0.532
AhMYB279	AHE.Chr27.837	299	34.13	6.32	58.34	63.28	−0.701
AhMYB280	AHE.Chr27.860	405	43.36	5.98	49.32	68.2	−0.439
AhMYB281	AHE.Chr27.862	405	43.36	5.98	49.32	68.2	−0.439
AhMYB282	AHE.Chr28.1	407	45.11	6.46	38.41	76.73	−0.546
AhMYB283	AHE.Chr28.1005	309	35.27	6.32	53.6	63.75	−0.674
AhMYB284	AHE.Chr28.1663	479	52.26	5.8	66.83	69.48	−0.639
AhMYB285	AHE.Chr28.1740.1	371	41.26	8.54	50.64	68.36	−0.68
AhMYB286	AHE.Chr28.1746	366	42.15	6.73	56.42	63.42	−0.891
AhMYB287	AHE.Chr28.1861	405	45.44	5.27	50.19	73.75	−0.637
AhMYB288	AHE.Chr28.1864	245	27.27	9.1	57.7	58.61	−0.81
AhMYB289	AHE.Chr28.2084	549	59.67	5.06	46.54	70.73	−0.504
AhMYB290	AHE.Chr28.313	380	40.87	9.49	44.36	60.82	−0.722
AhMYB291	AHE.Chr28.386	90	10.9	8.2	73.1	59.56	−1.079
AhMYB292	AHE.Chr28.453	273	30.44	9.17	47.98	75.46	−0.438
AhMYB293	AHE.Chr28.596	276	31.08	5.52	43.1	67.54	−0.756
AhMYB294	AHE.Chr28.645	380	40.82	9.59	43.54	59.79	−0.721
AhMYB295	AHE.Chr28.832	423	47.86	7.36	53.99	65.22	−0.982
AhMYB296	AHE.Chr28.848	260	27.79	7.7	48.55	75.15	−0.53
AhMYB297	AHE.Chr28.959	377	40.46	5.77	52.01	71.46	−0.389
AhMYB298	AHE.fragScaff_scaffold_206_pilon.54	373	41.41	6.9	59.87	84.96	−0.364

### 2.8 Determination of MDA Content

Samples (0.5 g) from different jackfruit varieties subjected to low‐temperature stress were homogenized in 1 mL of freezing extraction buffer containing 5.0‐mmol/L phosphate buffer (pH 5.7) and 8% polyvinylpyrrolidone. After centrifugation at 10,000 rpm for 4 min at 10°C, the supernatant was collected for enzyme activity determination. The MDA content was measured at 532 nm using a spectrophotometer, and nonspecific turbidity was corrected by subtracting the absorbance at 600 and 450 nm. Each experiment was replicated thrice.

### 2.9 Determination of CAT and Superoxide Dismutase (SOD) Activities

Samples (0.5 g) from different jackfruit varieties subjected to low‐temperature stress were homogenized in 1 mL of frozen extraction buffer containing 5.0‐mmol/L phosphate buffer (pH 5.7) and 8% polyvinylpyrrolidone. After centrifugation at 10,000 rpm for 4 min at 10°C, the supernatant was collected for enzyme activity determination. SOD activity was measured at 560 nm using spectrophotometry, and proline content was measured at 450 nm. Each experiment was replicated thrice.

### 2.10 Statistical Analysis

Gene expression data were analyzed using one‐way analysis of variance followed by Duncan's test to determine significant differences. Data were analyzed using SPSS Statistics 16.0 (SPSS Inc., Chicago, IL, USA) (Student's *t* test, **p < 0.01, ***p < 0.001, ****p < 0.0001). GraphPad Prism 9 (GraphPad Software, La Jolla, CA, USA) was used for graphical display.

## 3 Results

### 3.1 Identification and Phylogenetic Analysis of MYB Genes in *A. heterophyllus*

**FIGURE 1:**
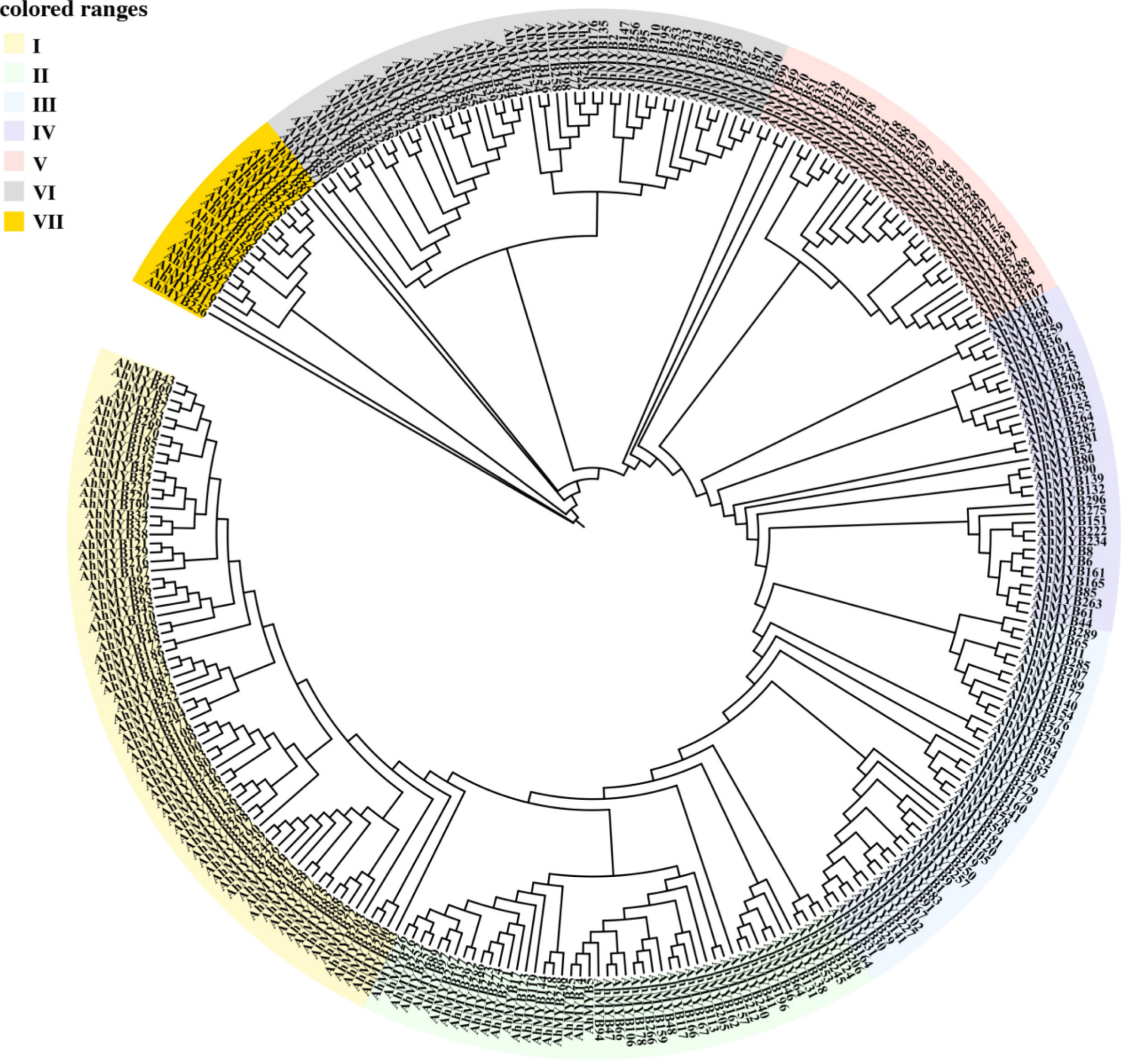
Phylogenetic tree analysis of MYB proteins from *Artocarpus heterophyllus* . The seven subgroups are represented by different colors: cream yellow for subgroup I, mint green for subgroup II, ice blue for subgroup III, taro for subgroup IV, light blue for subgroup V, gray for subgroup VI, and bright yellow for subgroup VII.

The “S10” *A. heterophyllus* genome was scanned, and 298 MYB genes were identified using BLAST searches with *Arabidopsis* and rice MYB genes. The MYB genes were annotated according to their position on the chromosome. To preliminarily investigate the function of MYB genes, the basic physicochemical properties of MYB proteins were analyzed using an online tool (Table 1). The number of amino acids (aa) in the MYB family protein sequences ranged from 52 (AhMYB210) to 1384 (AhMYB265), the theoretical isoelectric points (pI) ranged from 4.47 (AhMYB218) to 10.14 (AhMYB75), and the molecular weights ranged from 6.24 to 155.44 kDa. The instability indexes of 22 MYB proteins were less than 40, indicating they were stable, whereas those of the remaining 276 MYB proteins were greater than 40, classifying them as unstable. The phylogenetic relationships divided the AhWRKY gene family into seven subgroups (I–VII), with subgroup I containing the most members (72 AhWRKY genes) and subgroup VII the fewest (19 AhWRKY genes) (Figure 1).

### 3.2 Gene Structure and Conserved Motif Analysis

**FIGURE 2:**
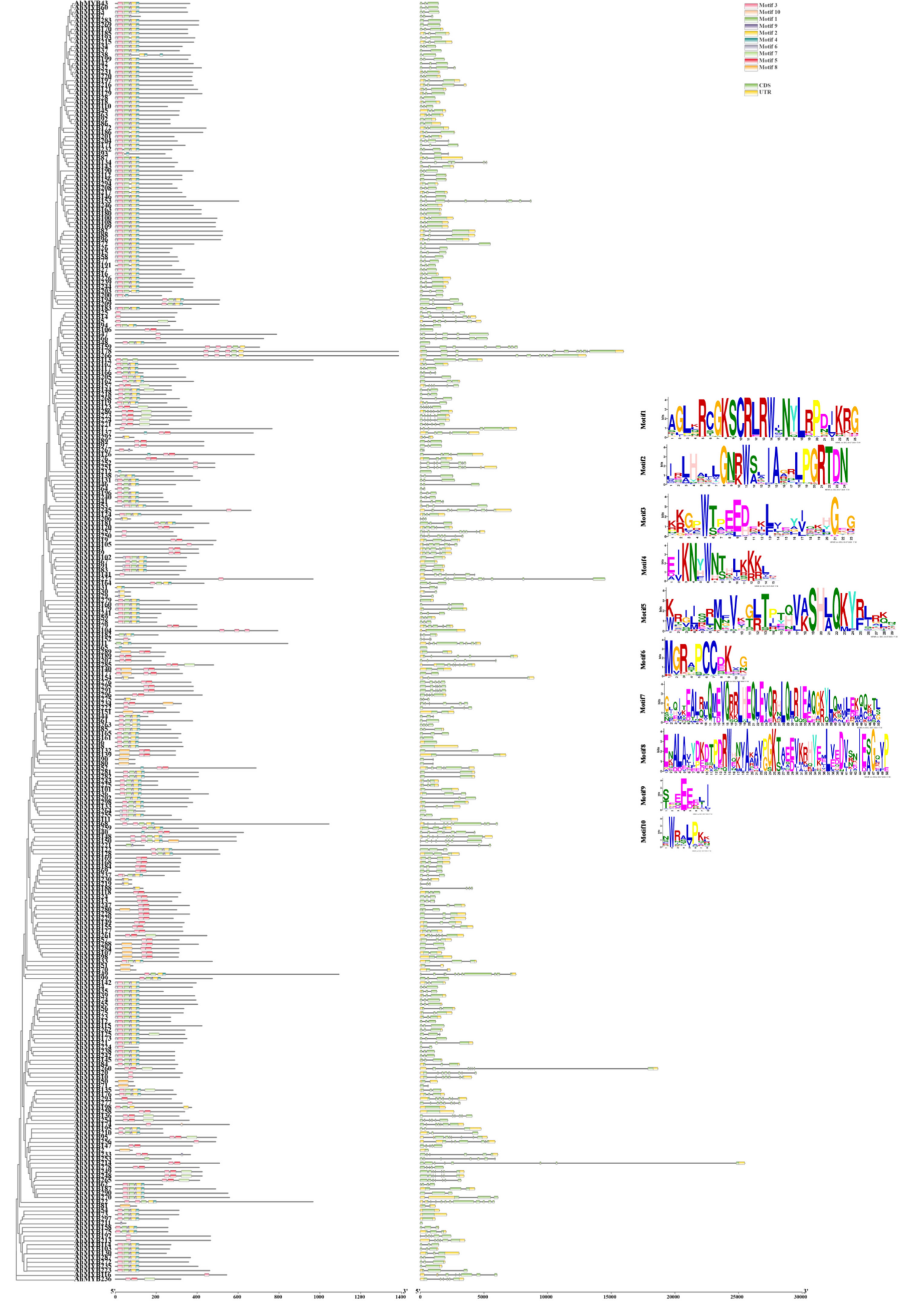
Gene structure, conserved motifs, and protein structure of the 298 AhMYB proteins.

Analysis of protein motifs, coding DNA sequences (CDSs), untranslated regions (UTRs), and gene structure of AhMYB genes revealed 10 distinct conserved motifs (Figure 2). Almost all AhMYB proteins contained Motif 3, indicating a high degree of conservation. In addition to the common conserved residue Trp (W), other conserved residues included Lys (K), Arg (R), Thr (T), Leu (L), Gly (G), Glu (E), Val (V), Asp (D), and Pro (P). Less than 25% of AhMYB proteins contained only one to two conserved motifs. Motif 10 was present in less than 10% of AhMYB proteins (Data S2: Table S2). Motifs 1, 3, and 10 were located near the N‐terminal, while Motifs 7 and 8 were positioned near the C‐terminal. AhMYB proteins in the same subfamily exhibited similar conserved domains, suggesting that they have similar functions. Analysis of gene structure is helpful to understand the function and regulation mechanism of gene expression, which is of great significance in biological research. The number of introns in the AhMYB genes ranged from 0 to 11, with 7 of 298 AhMYB genes containing no introns and 28 containing only one intron (Data S2: Table S2). AhMYB22 displayed the highest number of introns (18 introns), and approximately 71% of AhMYB genes had no more than two introns, indicating that the number of introns in the AhMYB family was limited.

### 3.3 Chromosomal Location Analysis

**FIGURE 3:**
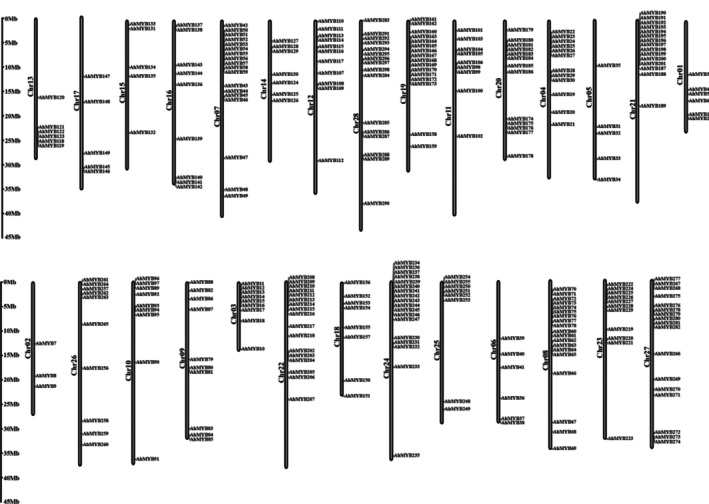
Chromosomal localization of MYB genes in jackfruit.

Using the annotated *A. heterophyllus* genome as a reference, the chromosomal locations of AhMYB genes were identified. The chromosomal distribution of AhMYB genes was mapped using TBtools (Figure 3). The *A. heterophyllus* genome contains 28 chromosomes, with chromosome 28 being the largest (44.1 Mb) and chromosome 3 being the smallest (14.6 Mb). The 298 AhMYB genes were distributed irregularly across all chromosomes, with the most AhMYB genes on chromosome 8 (19), followed by chromosomes 7 and 24 (18) and 22 (17). In contrast, fewer AhMYB genes were found on chromosomes 13 (six), 1 (six), 15, and 17 (five). The fewest AhMYB genes were found on chromosome 2, with only three AhMYB genes. The dense clusters of AhMYB genes on chromosomes 8 (19), 7, and 24 (18) suggest that these regions could represent evolutionary hot spots for the expansion of the MYB family in *A. heterophyllus* .

### 3.4 Synteny Analysis of MYB Genes in *A. heterophyllus*

**FIGURE 4:**
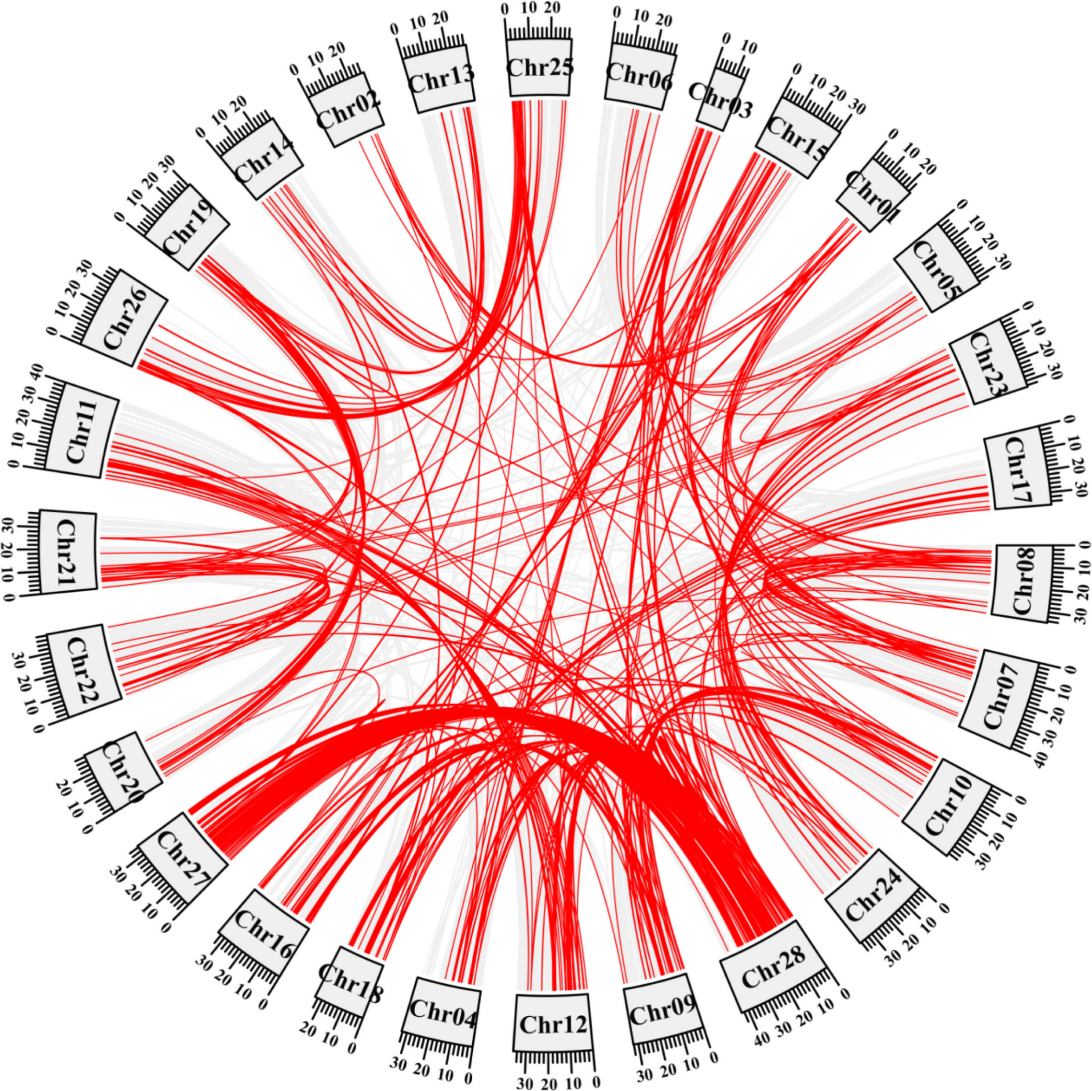
Collinearity of MYB genes in jackfruit.

MCScanX was used to analyze homology and determine the collinear gene pairs within MYB genes in *A. heterophyllus* . A total of 1439 collinear gene pairs were identified among AhMYB genes, with tandem duplication events observed across all 28 chromosomes of *A. heterophyllus* (Figure 4). In addition, several collinear gene pairs among AhMYB genes on chromosomes 27 and 28 of *A. heterophyllus* were observed, suggesting that AhMYB genes are functionally conserved during evolution, and tandem duplication events may be the main route of evolution.

### 3.5 Prediction of *cis*‐Acting Elements of MYB Genes in *A. heterophyllus*

**FIGURE 5:**
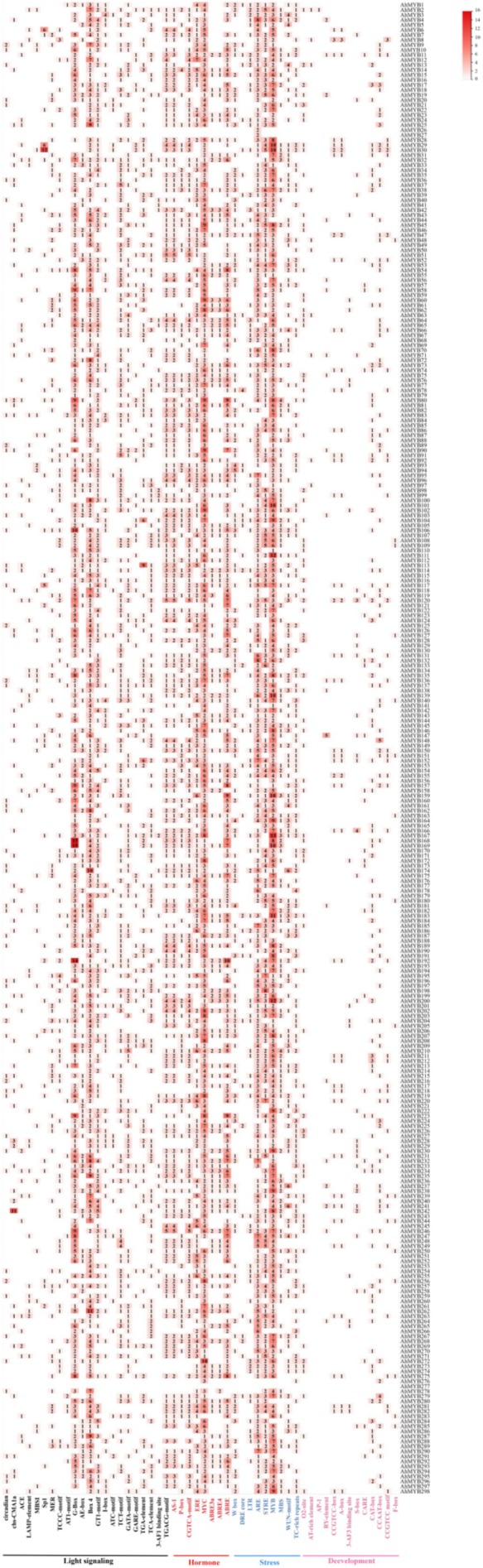
Analysis of the cis‐acting elements of MYB gene promoters in jackfruit. The number of elements involved in photoresponse, hormone response, abiotic stress, and growth and development is different. The color shades and numbers in the squares indicate the number of promoters.

Promoter *cis*‐acting elements are important components of gene expression and are involved in plant growth and development, as well as hormone and stress responses (Yamaguchi‐Shinozaki and Shinozaki 2005). To further investigate functional differences among AhMYB gene promoters, *cis*‐acting elements were analyzed using PlantCARE based on the 2‐kb upstream sequences from the initiation codons of 298 AhMYB genes (Figure 5). The identified *cis*‐acting elements were divided into four groups: light signaling response (22), hormonal response (8), abiotic stresses (9), and development elements (14). Hormone response elements (ABREs), JA response elements (MYC), and ethylene response elements (ERE) accounted for the largest proportion. MYB (CTAACCAAG), anaerobic response (ARE), and stress response (STRE) components comprised the largest proportion of stress response components. In addition, growth and development‐related *cis*‐acting elements included the commonly observed meristem‐specific expression site (CAT‐box) and seed‐specific regulatory element (RY‐elements). These results suggest that AhMYB genes are involved in light, hormone regulation, and stress response in *A. heterophyllus* and may operate through various regulatory modes.

### 3.6 Expression Profile Analyses of MYB Genes in *A. heterophyllus* Under Cold Stress

**FIGURE 6:**
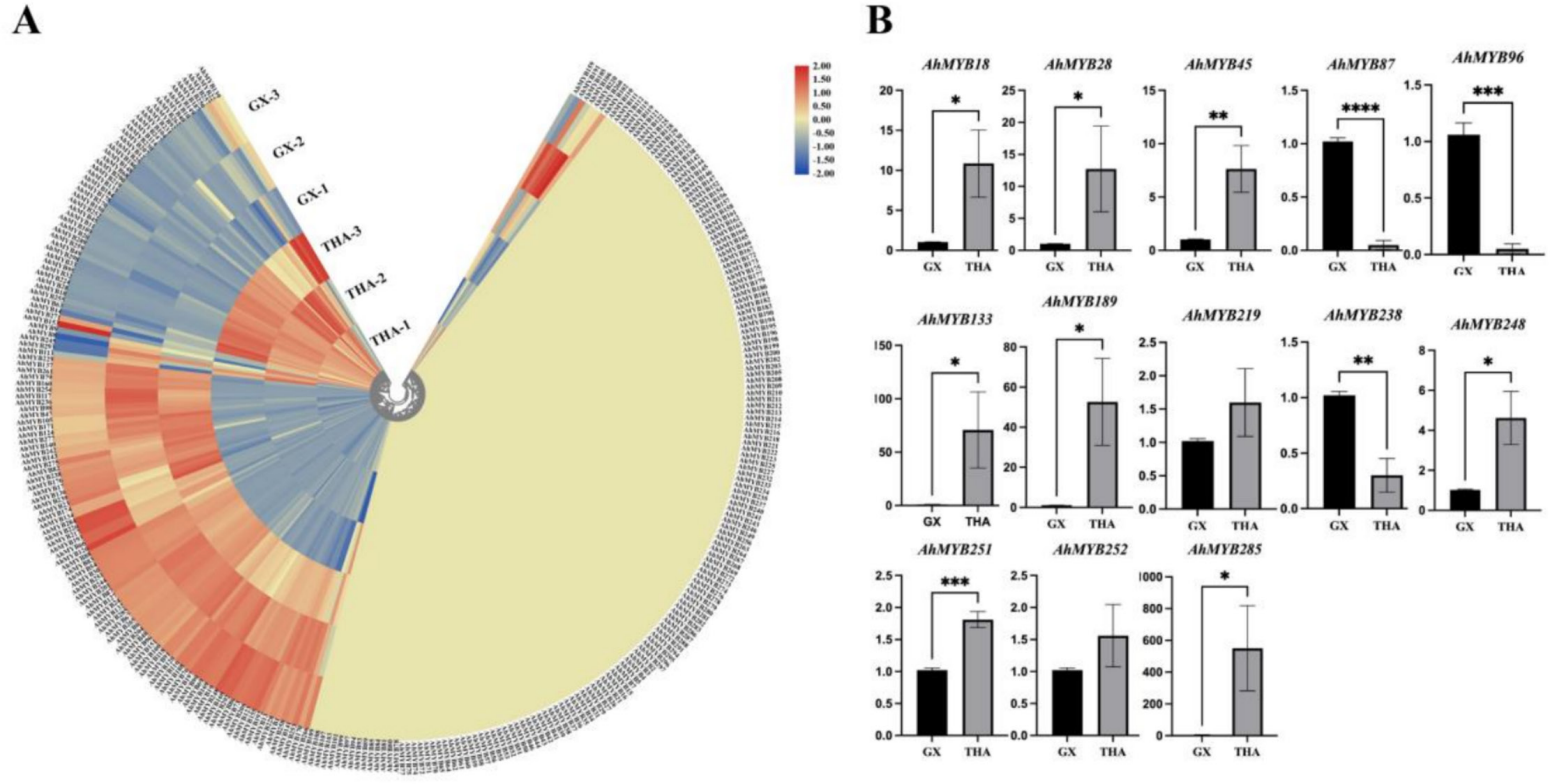
Expression pattern analysis of MYB genes in jackfruit under low‐temperature stress. (A) Heat map analysis of MYB genes in jackfruit varieties under low‐temperature stress. (B) qPCR expression patterns of differentially expressed genes under low‐temperature stress. *p < 0.1, **p < 0.01, ***p < 0.001, and **** *p* < 0.0001.

Low temperature is a common environmental stress factor that restricts the geographical distribution of plants and affects their growth and development (Yuanyuan et al. 2009). To explore the expression pattern of AhMYB genes in different jackfruit varieties under low‐temperature stress, we used our previously published transcriptome data (Ma et al. 2024) to illustrate their expression patterns. The results showed that 157 AhMYB genes (FPKM < 1) were almost not expressed in the transcriptomic data of *A. heterophyllus* (Figure 6A). In the expressed genes, 90 genes were highly expressed in the GX variety, and 51 genes were highly expressed in THA. These results suggest that AhMYB genes may play an important role in resistance to cold stress in different jackfruit varieties. It is worth noting that under the low‐temperature stress, AhMYB genes (AhMYB18/28/45/87/96/133/189/219/238/248/251/252/285) were differentially expressed in different strains of jackfruit. The results indicated that the 13 AhDREB genes of GX might have stronger resistance to cold stress than those of THA.

To further verify the expression pattern of AhMYB genes under cold stress, we screened 13 AhMYB genes (AhMYB18/28/45/87/96/133/189/219/238/248/251/252/285) that were differentially expressed in the transcriptome and verified them using qPCR (Figure 6B). Genes such as AhMYB18/28/45/133/189/219/248/251/252/285 showed increased expression in the THA variety, whereas AhMYB87/96/238 exhibited lower expression. In addition, *cis*‐acting element analysis of AhMYB gene promoters revealed the presence of low‐temperature‐responsive (LTR) elements in response to cold stress, suggesting that AhMYB genes may play an important role in cold stress resistance.

### 3.7 Functional Analysis of GX and THA Under Cold Stress

**FIGURE 7:**
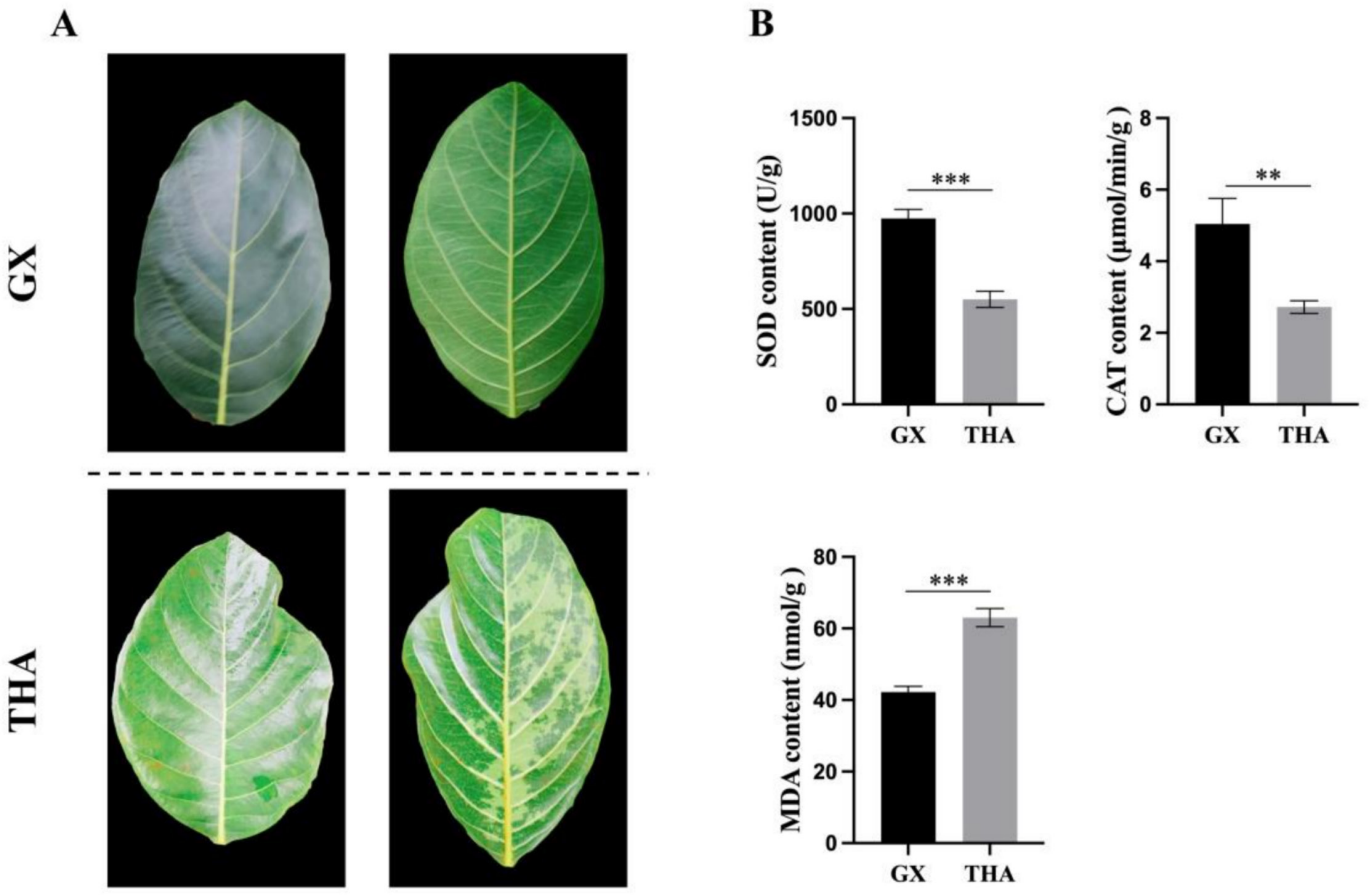
Determination of the physiological indicators of different jackfruit varieties. (A) GX and TAH phenotypes after cold stress. (B) SOD, CAT, and MDA levels in GX and TAH after cold stress *p < 0.1, **p < 0.01, ***p < 0.001, and ****p < 0.0001.

CAT and SOD are important antioxidant enzymes in plants that play a key role in the response of plants to oxidative stress, maintenance of reactive oxygen species (ROS) balance, and adaptation to adverse conditions (Azarabadi et al. 2017). An increase in MDA content directly indicates that the cell membrane system is under attack by ROS, leading to membrane structural damage. When plants are subjected to abiotic stress, the MDA content in plants usually increases significantly (Ayala et al. 2014). After exposure to low‐temperature stress, distinct water‐soaked spots appeared on THA leaves, indicating that GX leaves were less damaged. To explore the mechanism by which different jackfruit varieties respond to low‐temperature stress, we determined their contents of CAT and SOD after low‐temperature stress (Figure 7). The results showed that CAT and SOD levels in GX were significantly higher than those in THA. Moreover, we determined the MDA content in different jackfruit varieties and found significantly higher levels in THA than those in GX. These results further indicate that GX exhibits greater cold resistance than THA.

## 4 Discussion

### 4.1 MYB Genes Are Evolutionarily Conserved in *A. heterophyllus*

MYB, as one of the largest families of TFs in plants, contains a highly conserved DNA‐binding domain, which enables it to specifically bind to downstream target sequences, thereby regulating gene expression levels and affecting plant growth and development and response to stress (Pireyre and Burow 2015). Klempnauer et al. (1982) identified the first MYB TF in avian myeloblastosis virus, which was named v‐MYB. MYB TFs were later identified in eukaryotes such as animals, plants, and fungi (Lipsick 1996). The maize ( *Zea mays* ) ZmMYBC1 gene, which regulates pigment synthesis, was the earliest identified MYB‐like TF in plants (Paz‐Ares et al. 1987). Subsequently, a large number of MYB TFs have been cloned and identified in plants, where they play an important role in transcriptional regulation. In the current study, 298 nonredundant MYB genes were identified in *A. heterophyllus* S10. The number of MYB genes detected in *A. heterophyllus* was lower than that in *Stevia rebaudiana* (374) (Chen et al. 2024) and *Gossypium hirsutum* (524) (Salih et al. 2016) and higher than that in *S. rebaudiana* (127) (Chen et al. 2024) and *Pyrus bretschneideri* (231) (Li et al. 2016). These results indicate that MYB genes differentially respond to environmental stress, adaptive changes, and genetic diversity throughout evolution. Analysis of the basic physicochemical properties of AhMYBd proteins showed significant differences in the molecular weight of proteins encoded by different genes, consistent with previous studies (Feng et al. 2017). Conserved motif analysis showed that almost all AhMYB genes contained Motif 3, and members of the same subfamily in the phylogenetic tree shared similar Motif 3 patterns and gene structure. In addition, most AhMYB genes contained only two introns, which are consistent with the fact that most MYB genes in higher plants contain only two introns (Abbas et al. 2021). Chromosome localization analysis showed that the 298 AhMYB genes were unevenly distributed across 28 chromosomes, with small gene clusters also observed. Collinear analysis revealed 1439 collinear gene pairs among AhMYB genes. It is speculated that segmental replication and tandem repeat events are the main modes of AhMYB gene expansion in *A. heterophyllus* , which may enhance the adaptability of the plant to different environmental conditions (Schilling et al. 2020).

### 4.2 MYB Genes May Be Involved in Cold Stress Resistance in *A. heterophyllus*

Under abiotic stress, plants initiate a series of stress responses that activate defense mechanisms to cope with environmental changes (Chaudhry and Sidhu 2022). The signal transduction pathway of plant cells in response to cold stress is complex. To date, several MYB TFs have been confirmed to participate in the process of plant response to cold stress. Agarwal et al. (2006) found that under low‐temperature conditions, the *Arabidopsis* TF AtMYB15 regulates cold resistance by binding to the MYB recognition sequences of CBF (CBF1, CBF2, and CBF3) gene promoters and inhibiting the expression of CBF genes. Abubakar et al. (2022) identified AvMYB48, AvMYB97, AvMYB8, and AvMYB4 as potential stress response genes by analyzing MYB expression in *Apocynum venetum* at low temperatures. In the phenylpropane metabolic pathway of *Dendrobium officinale* under freezing stress, DoMYB97, DoMYB39, fructosyltransferase, and zinc finger protein TFs were significantly affected by freezing and freezing recovery (Zhan et al. 2022). The *cis*‐acting elements of promoters play an important biological role in regulating plant gene expression by interacting with TFs (Otsuki and Yamamoto 2020). In the present study, analysis of the promoter *cis*‐acting elements of MYB genes revealed that AhMYB genes contain a large number of promoter *cis*‐acting elements in response to stress, some of which contained LTR elements. Therefore, we combined the transcriptomic data of different jackfruit varieties under low‐temperature stress to explore whether MYB genes play a role in the cold resistance of jackfruit. The results showed that most AhMYB genes were highly expressed in GX. To further verify this result, differentially expressed genes were screened and verified using qPCR. The expression trend of these differentially expressed AhMYB genes was consistent with the transcriptome results. Thus, it is speculated that AhMYB genes may play an important role in the resistance of jackfruit to cold stress, with each member playing distinct roles in different jackfruit varieties. However, their specific roles require further validation. The results of this study provide new insights into the response mechanism of jackfruit to cold stress.

### 4.3 GX Experiences Reduced Low‐Temperature Stress‐Induced Damage by Regulating the Antioxidant Enzyme Levels

Plants encounter various stresses during growth. Under low‐temperature stress, the metabolic processes of plants produce ROS, which accumulate in large quantities within the plants and cause membrane lipid peroxidation (Airaki et al. 2012). This effect leads to the gradual accumulation of MDA, which can exacerbate the damage to biological membranes within plants and severely hinder or even disrupt various metabolic activities (Hasanuzzaman et al. 2021). SOD and CAT are important antioxidant protective enzymes in plant cells, and their contents are commonly used physiological indicators of stress tolerance (Hasanuzzaman et al. 2011). MDA, as an indicator of lipid peroxidation, is often used to measure the degree of damage to plants. Yan et al. (2010) found that the MDA levels in *Forsythia suspensa* at low temperatures increased by 66.7% compared with those at normal temperatures. After low‐temperature treatment, SOD, CAT, and MDA levels in two strawberry varieties (cv. Zoji and Toyonaka) were all higher than those in the control (Luo et al. 2011). After long‐term exposure to cold treatment, the SOD enzyme activities of three citrus varieties (*Citrus unshiu*, *Citrus sinensis* , and *Citrus limon* ) all increased significantly (Mohammadian et al. 2012). After being subjected to low‐temperature stress, water‐soaked spots appeared on the leaves of THA. To further explore the mechanism by which different jackfruit varieties respond to low‐temperature stress, we determined their SOD, CAT, and MDA levels. The results showed that SOD and CAT levels in GX were significantly higher than those in THA. However, MDA levels in GX were significantly lower than those in THA. Therefore, it can be inferred that GX may respond to external low‐temperature stress by regulating the activity of its antioxidant enzymes, thereby increasing its stress resistance. This result is consistent with the mechanism by which wheat (Hou et al. 2010) ( *Triticum aestivum* ) and strawberries (Luo et al. 2011) (*Fragaria* × *ananassa*) respond to low‐temperature stress.

## Author Contributions

Conceived the project: Pengjin Zhu and Hailan Zhou. Experiments data collection: Xiangwei Ma, Jianjun Liang, and Chenxin Yi. Analysis and interpretation of results: Chenxin Yi, Weiyan Ye, and Zhuangmin Wei. Draft manuscript preparation: Xiuguan Tang, Qiqi Song, and Shengli Tang. All authors reviewed and approved the final manuscript.

## Funding

This research was supported by the Special Project for Basic Scientific Research Business of Guangxi Academy of Agricultural Sciences (GNK 2025YP128, GNK 2026YT021, GNK2026YP054), and the vanguard team of the specialty fruit industry GNKM (202504‐03).

## Supporting information


**Data S1:** Supporting information.

**Data S2:** Supporting information.

**Data S3:** Supporting information.
